# Studying root–environment interactions in structured microdevices

**DOI:** 10.1093/jxb/erad122

**Published:** 2023-04-12

**Authors:** Christian-Frederic Kaiser, Alessia Perilli, Guido Grossmann, Yasmine Meroz

**Affiliations:** Institute of Cell and Interaction Biology, Heinrich-Heine-University Düsseldorf, Universitätsstrasse 1, D-40225 Düsseldorf, Germany; CEPLAS - Cluster of Excellence on Plant Sciences, Heinrich-Heine-University Düsseldorf, Universitätsstrasse 1, D-40225 Düsseldorf, Germany; School of Plant Sciences and Food Security, Tel Aviv University, Tel Aviv, Israel; Institute of Cell and Interaction Biology, Heinrich-Heine-University Düsseldorf, Universitätsstrasse 1, D-40225 Düsseldorf, Germany; CEPLAS - Cluster of Excellence on Plant Sciences, Heinrich-Heine-University Düsseldorf, Universitätsstrasse 1, D-40225 Düsseldorf, Germany; School of Plant Sciences and Food Security, Tel Aviv University, Tel Aviv, Israel; Centre for Research in Agricultural Genomics (CRAG), Spain

**Keywords:** Biosensors, complex conditions, 3D printing, lab-on-a-chip, live imaging, microfluidics, root–microbe interactions, synthetic environments

## Abstract

When interacting with the environment, plant roots integrate sensory information over space and time in order to respond appropriately under non-uniform conditions. The complexity and dynamic properties of soil across spatial and temporal scales pose a significant technical challenge for research into the mechanisms that drive metabolism, growth, and development in roots, as well as on inter-organismal networks in the rhizosphere. Synthetic environments, combining microscopic access and manipulation capabilities with soil-like heterogeneity, are needed to elucidate the intriguing antagonism that characterizes subsurface ecosystems. Microdevices have provided opportunities for innovative approaches to observe, analyse, and manipulate plant roots and advanced our understanding of their development, physiology, and interactions with the environment. Initially conceived as perfusion platforms for root cultivation under hydroponic conditions, microdevice design has, in recent years, increasingly shifted to better reflect the complex growth conditions in soil. Heterogeneous micro-environments have been created through co-cultivation with microbes, laminar flow-based local stimulation, and physical obstacles and constraints. As such, structured microdevices provide an experimental entry point into the complex network behaviour of soil communities.

## Teaser

How plant roots cope with the soil complexity and integrate heterogeneous conditions into development, defence, and metabolism remains unclear. Structured microfluidic devices now enable controlled generation of complex microenvironments for microscopy-based root studies.

## Introduction

The first point of contact of plants with their soil environment is their root system, and as such root function has critical implications for the whole organism, decisive for nutrient uptake, carbon sequestration, and interaction with microorganisms (Fita *et al*., 2015; Barkaoui *et al*., 2016; Fry *et al*., 2018; Robinson *et al*., 2018; Ober *et al*., 2021), while also rapidly responding and plastically acclimating their architecture to various stress factors (Gruber *et al*., 2013; Gilroy *et al*., 2014; Keinath *et al*., 2015; Stanley *et al*., 2018; Correa *et al*., 2019). Roots shape the soil environment through the establishment of the rhizosphere, by increasing the bioavailability of mineral nutrients, influencing soil structure, and providing a specific carbon-rich niche for adapted microbial communities (Guyonnet *et al*., 2018; Canarini *et al*., 2019; Correa *et al*., 2019; Lucas *et al*., 2019).

**Fig. 1.**
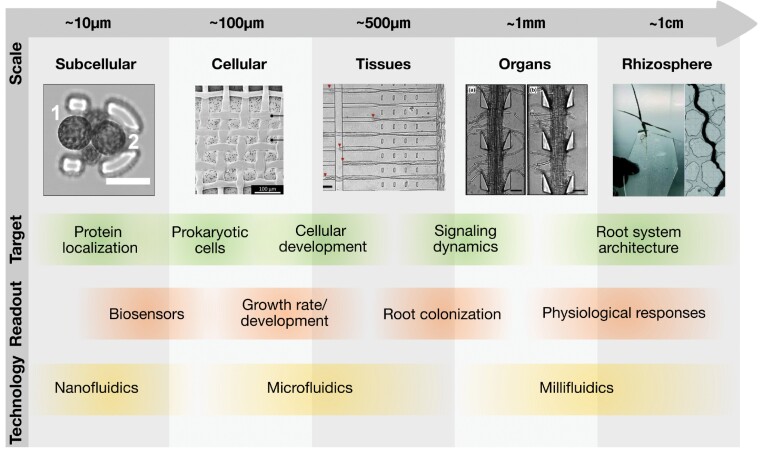
Relative scale of microfluidics applications, targets, and readouts. Microfluidic devices can be designed to address questions at multiple scales, from subcellular (~µm) to the rhizosphere (~cm), targeting a variety of processes, from protein localization to root system development. Illustration images, from left to right: Sakai *et al*., 2019; Finkbeiner *et al*., 2022; Yanagisawa *et al*., 2017; Stanley *et al*., 2018, the Open Access licence covering this article does not apply to this image; Aufrecht *et al*., 2022. A detailed list of devices, including time scales and length scales, appears in Table 1. Images are reproduced with permission of their respective copyright holders.

Root–environment interactions are characterized by spatial and temporal heterogeneity stemming from the level of a single soil particle up to the whole root system, with fluctuations from seconds to days (Aleklett *et al*., 2018). The biological processes involved act on drastically different spatial scales—from subcellular protein function to cellular processes such as the perception of environmental stimuli, second messenger signalling to organ development, and restructuring of the root system architecture (Fig. 1) (Kazan, 2013; Fichman and Mittler, 2020; Karlova *et al*., 2021). Elucidating root traits, and how roots integrate sensory information leading to a strategic growth pattern, requires a fundamental understanding of the mechanisms underlying the integration of the complex soil environment into plant physiology and development from the micro- to macroscale and from seconds to days.

The challenge of root analysis across spatial and temporal scales has been met with the development of sophisticated root phenotyping systems, allowing the coupling of genotypic profiles to root phenotypes (Clark *et al*., 2020). These approaches aim to overcome the major limitation of accessing the ‘hidden half’ of plants (Atkinson *et al*., 2019), which is the visual inaccessibility of the soil matrix, that traditionally requires extracting the root system. As such, approaches such as the ‘GloRoot’, bioluminescence-based *in situ* tracking of root development in thin-soil sheets, such as rhizotrons (Rellán-Álvarez *et al*., 2015), or the utilization of microcomputed tomography (µ-CT) (Atkinson *et al*., 2019; Teramoto *et al*., 2020), have enabled imaging root systems in their native environment. While these systems allow minimally invasive access to root system architecture and high-throughput phenotyping, the spatial and temporal resolution is limited, and cannot tap into microscale or rapid processes, critical to fully grasp the complexity of the rhizosphere. Multifaceted approaches that include high-resolution imaging techniques (Clark *et al*., 2020), genetic tools such as genetically encoded biosensors (recently reviewed in Sadoine *et al*., 2021), and manipulation of environmental conditions, remain incompatible with a native soil environment. This is due to the fact that traditional microscopic techniques cannot be used and, furthermore, environmental conditions cannot be precisely controlled. Commonly, microscopic studies of root systems have therefore turned to accessible but artificially homogeneous agar-based or hydroponic growth of plants (Gregory *et al*., 2009; Asao, 2012). While allowing high-resolution imaging, agar-based setups typically lack the structural, chemical, and biotic ­diversity posed by a native soil environment. Therefore, studies of root systems compromise between having a controlled system, imaging capabilities, or the ability to mimic soil-like conditions.

In recent years, there has been a transfer of microfluidic technology (Box 1) from the fields of chemical analysis, medical diagnostics, and mammalian cell biology into plant science (Yanagisawa *et al*., 2021). The development of new structured microdevices has met the challenge of stimulating and imaging roots at high spatiotemporal resolution. Microfluidics harnesses the laminar flow-dominated fluid dynamics (Box 2) of minuscule volumes, and possibilities of rapid design and prototyping to generate defined controlled environments. Since the 2010s with the development of the first *Arabidopsis thaliana-*adapted approaches (Meier *et al*., 2010; Grossmann *et al*., 2011; Parashar and Pandey, 2011; Busch *et al*., 2012) micro- to milli-fluidic devices have advanced in the range of accessible species, precise environmental control, possibilities of co-cultivation with microbial communities, and available scaffolds (Fig. 1). Novel technologies such as 3D printing now move the boundaries of accessibility and turn an originally exotic technique into a potential backbone for an advanced ‘soil-on-a-chip’ toolkit for root–environment studies, a concept proposed by Stanley *et al*. (2016), that not only improves existing capabilities but also enables experimental designs which were not feasible until now. An overview of root-adapted microfluidic devices, including details about the relevant time scales, length scales, and number of samples, is shown in Table 1.

**Table 1.** Overview of root-adapted microfluidic devices displaying plants per device, spatial resolution, and reported time span of the specimen on the device

Device	Reference	Organism	Plants per device	Resolution	Time span
Multi-laminar flow device	Meier *et al*. (2010)	*A. thaliana*	1	µm	1 d
Plant-in chip	Parashar and Pandey (2011)	*A. thaliana*	8	μm	4 d
RootChip	Grossmann *et al*. (2011)	*A. thaliana*	8	μm	3 d
RootArray	Busch *et al*. (2012)	*A. thaliana*	64	μm	3 d
RootChip16	Jones *et al*. (2014)	*A. thaliana*	16	μm	5 d
dfRootChip	Stanley *et al*. (2018)	*A. thaliana*	5	μm	3 d
Plant Array Chip	Park *et al*. (2017)	*A. thaliana*	400	mm	10 d
TRIS Device	Massalha *et al*. (2017)	*A. thaliana*	9	μm	5 d
EcoFab	Sasse *et al*. (2018)	*B. dystachion*	1	μm	21 d
vRootChip	Fendrych *et al*. (2018)	*A. thaliana*	4	μm	3 d
Porous network device	Aufrecht *et al*. (2017)	*A. thaliana*	1	μm	4 d
Foldable Chip Array	Song *et al*. (2019	*N. tabacum*	4	μm	10 d
Petaloid Root-growth chip	Chai *et al*. (2019)	*O. sativa*	1	mm	11 d
RootChip-8S	Denninger *et al*. (2019)	*A. thaliana*	8	μm	5 d
RMI-chip	Noirot-Gros *et al*. (2020)	*P. tremuloides*	6	μm	10 d
Coverslip-based microfluidic device	Singh *et al*. (2021)	*A. thaliana*	3	μm	5 d
Rhizosphere-on-a-chip	Aufrecht *et al*. (2022)	*B. dystachion*	1		12 d
RootTrapr	Suwanchaikasem *et al*. (2022)	*C. sativa*	1	mm	14 d
Microfluidic device	Dai *et al*. (2022)	*O. sativa*	1	μm

**Box 1. Fabrication and production of microdevices**

Microfluidic devices are typically fabricated by casting PDMS [poly(dimethylsiloxane)] into a mould carrying the specific design features, such as microchannels or microchambers, needed for the experiment, a process called soft lithography (Xia and Whitesides, 1998). The mould is most commonly manufactured through photolithography techniques, which provide extremely high feature resolution at a sub-micrometre scale. This technique, however, has the disadvantage of being costly and requiring specific expertise.

Currently, soft lithography (called ‘soft’ because it uses elastomeric materials, such as PDMS) is the most common solution for the fabrication of microfluidic devices, even though it has been pointed out that PDMS has its own drawbacks—its processing requires relatively long times, it is difficult to seal, and it swells when exposed to many organic solvents. The identification of both materials and procedures that might lead to affordable, upscalable production of microfluidic devices, though, is still an open challenge (Battat *et al*., 2022).

Photolithography can be replaced by 3D printing for the fabrication of the mould—3D printers are not only more affordable and accessible (lowering the setup cost from US$80 000 to US$1000–20 000) but can also provide a fairly high resolution, ranging from tens to hundreds of micrometres. 3D printing also offers an advantage in terms of turnaround time, which can be <2 h, compared with ~24 h for soft lithography (Bhattacharjee *et al*., 2016).

Furthermore, microfluidic devices have been fabricated by cutting features from a silicon sheet and enclosing it between two coverslips (Bertrand-Rakusová *et al*., 2020); in this case, the spatial resolution of the features is not comparable with moulded PDMS, but still satisfactory for certain applications. Alternatively, commercial pre-designed microfluidic chambers can be bought for reasonable prices, but have the drawback of not being customizable in the design.

**Box 2. Microfluidic flow dynamics/parameters**

The term microfluidics indicates both the science that studies the behaviour of fluids in micro-sized channels and the technological fabrication of micro-devices, embedding chambers, and channels (typical dimensions on the order of tens to hundreds of micrometres) for the confinement or flow of fluids at the microscale (typical volumes on the order of 10^–3^–10^–6^ μm^–3^). The use of fluids in such conditions offers a number of advantages, for example the ability to use small, controlled amounts of reagents or samples, high resolution and sensitivity in both the measurements and the manipulation of the samples, low costs, and short times for analysis, allowing high throughput. Furthermore, fluids at such scales show a behaviour very different from at the macro-scale, thanks to the typically small Reynolds numbers involved: the Reynolds number is a dimensionless quantity, which predicts flow patterns, related to the density and viscosity of the fluid and to the typical velocity and characteristic dimension of the flow. Systems with a high Reynolds number, typically macroscopic, exhibit turbulent flows, with particles moving irregularly. Systems with a low Reynolds number, typically microscopic, exhibit laminar flow, with particles moving in parallel in smooth straight trajectories. In this latter case, different fluids stay perfectly separated as mixing does not occur, thus allowing control of the location and movement of reagents, suspensions, or cells in the channels (see Figure for an explanatory sketch).

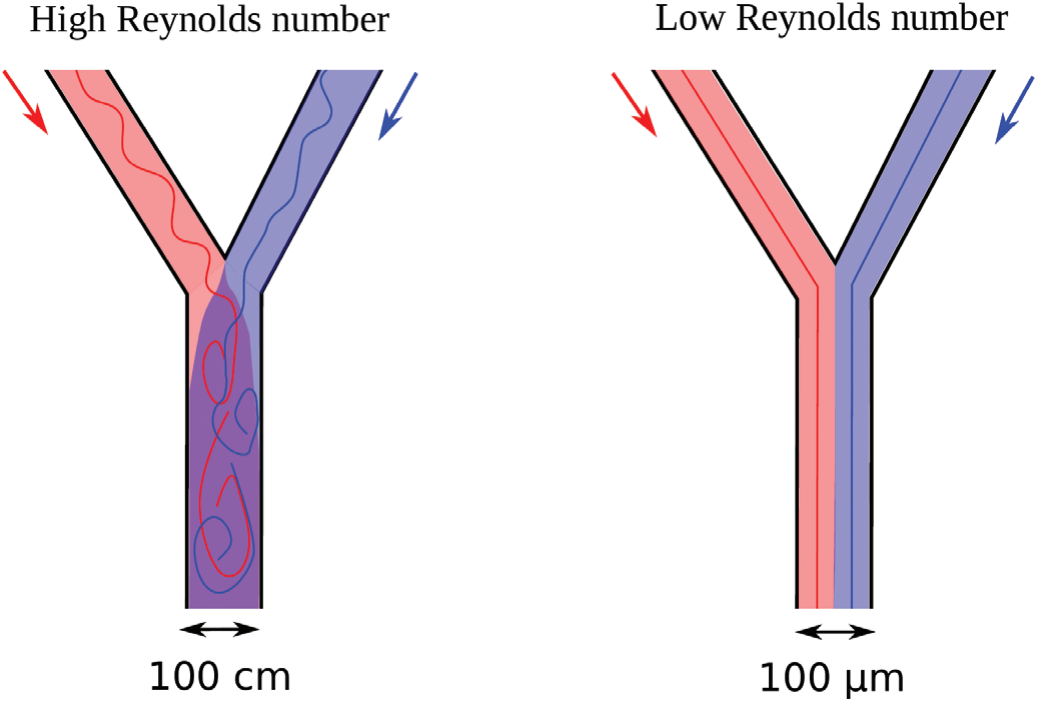

Turbulent versus laminar flows: in the left panel, two different fluids entering a middle channel from two sides give rise to a turbulent flow (high Reynold’s number), with the red and blue fluids mixing together. The same geometry in a microsized channel (right panel) would lead to a laminar flow with the two fluid components remaining perfectly separated (no mixing).

## Reflecting soil-like heterogeneity in structured microdevices

**Fig. 2.**
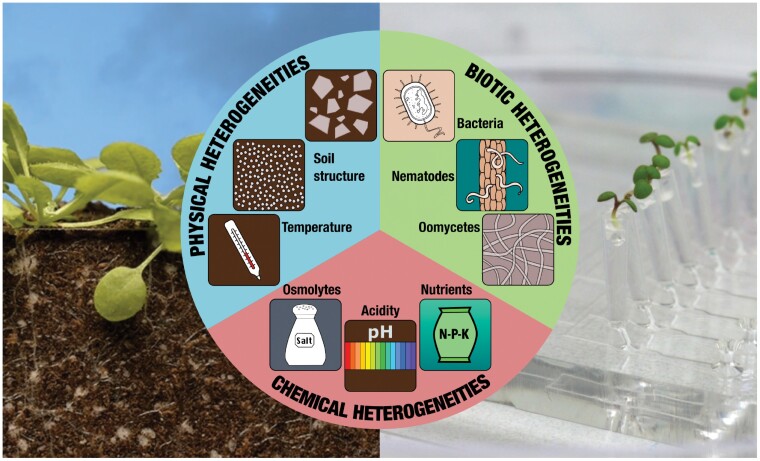
Schematic representation of environmental heterogeneities in the root–soil interplay—namely physical (soil structure and temperature), chemical (osmolytes, nutrients, acidity), and biotic (bacteria, nematodes, filamentous eukaryotes such as oomycetes). While these factors are entangled and not easily separable in real soil, microfluidics and soil-mimicking microdevices allow the control of each one of them and investigate the response of the root system with a precision ranging from the micro- to macro-scale and on time scales ranging from seconds to days. (Left image © Wim van Egmond, still from a time-lapse made in collaboration with Gerlinde De Deyn, Wageningen University and Research 2020. This image is reproduced with permission and is not covered by an Open Access licence).

**Fig. 3.**
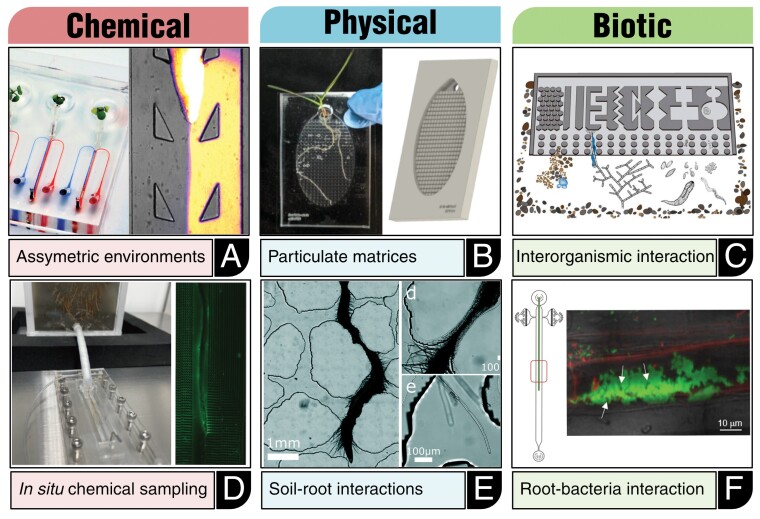
Range of chemical, physical, and biotic parameters of root–soil interactions addressed in structured microdevices. (A) Generation of asymmetric environmental conditions through bilaminar flow in the dual-flow RootChip (Stanley *et al*., 2018), with permission; the Open Access licence covering this article does not apply to this image. (B) Integration of a pillar-based matrix into a EcoFab to simulate the particulate soil matrix (Jabusch *et al*., 2021). (C) Assessing multi-organismic interaction between fungi and bacteria on a heterogenous soil-like chip (Mafla-Endara *et al*., 2021). (D) *In situ* imaging of ROS production of mature rice roots. Reprinted with permission from Dai *et al*. (2022). Diel fluctuation of extracellular reactive oxygen species production in the rhizosphere of rice. Environmental Science & Technology 56, 9075–9082. Copyright 2022 American Chemical Society, the Open Access licence covering this article does not apply to this image. (E) Root growth in a soil-like matrix (Aufrecht *et al*., 2022), and root colonization of *Populus tremoloides* by *Pseudomonas fluorescens* on the RMI chip (Noirot-Gros *et al*., 2020).

Soil, including the rhizosphere, represents the complex interplay of the lithosphere, hydrosphere, atmosphere, and biosphere (Totsche *et al*., 2010). This results in an environment that involves dynamic and spatially heterogeneous physical, chemical, and biological factors (Fig. 2). Soil-mimicking microdevices therefore need to account for the spatiotemporal heterogeneity of various stimuli of the soil environment (Fig. 3).

### Physical heterogeneity

Soil is a matrix of particulate matter including inorganic and organic compounds associated as suspended particles, colloids, and rock, which again define channels, pores, and aggregates of scales between micrometres and millimetres (Totsche *et al*., 2010). This matrix is filled with gaseous, liquid, and solid phases, and therefore presents a spatially heterogeneous environment with diverse mechanical properties, and associated fluid dynamics (Aleklett *et al*., 2018). Roots are responsive to mechanical stimuli associated with the soil structure by thigmotropic object avoidance and changes in branching patterns (Dunbabin *et al*., 2013; Kolb *et al*., 2017; Ponce *et al*., 2017; Correa *et al*., 2019).

‘Transparent soil’ technology has been developed in order to reconstitute soil structure in visually accessible experimental setups. This refers to optically water-like artificial particles utilized to form a soil-like particulate matrix. As such, the polymer Nafion, a synthetic ionomer, has been proven to be particularly useful due to its refractive index (RI) of *n*=1.228–1.310 (Bayani Ahangar *et al*., 2019), which closely matches the RI of water [and most experimentally used plant media; *n*(water)=1.333] and thus renders the material virtually invisible in liquid-filled cuvettes or chambers. Hence, Nafion particles can emulate soil particles, while minimizing shading or optical distortions during the microscopy of fluorescent biological specimens (Downie *et al*., 2012). Nafion matrices have been used not only to image root system development, but also to perform high-resolution confocal imaging of root–microbe interactions between *A. thaliana* and the enterobacterium *Escherichia coli*, allowing the capture of *in situ* microcolony formation (Downie *et al*., 2012). In the field of soil microbiology, Nafion particles have also been introduced into microfluidic setups to track the influence of fluctuating environmental conditions on bacterial communities through stable isotope labelling (Sharma *et al*., 2020). In a similar fashion, biopolymer-based hydrogels such as calcium/magnesium alginate have been used to track the root development of *Glycine max* and associated pH changes in the surrounding substrate (Ma *et al*., 2019). In comparison, hydrogel-based beads are more cost-efficient and customizable regarding their composition, while Nafion particles represent a reusable matrix of impenetrable particles of great shape diversity.

Root-adapted microfluidic devices did not initially hold particulate matrices of any kind, but rather utilized linear channels for guiding root growth, or utilized unstructured chambers (Meier *et al*., 2010; Grossmann *et al*., 2011; Parashar and Pandey, 2011; Gao *et al*., 2018; Massalha *et al*., 2019). A network of channels featuring a variety of different structural elements was first employed for the investigation of the tip growth of *Camellia* pollen tubes, allowing the measurement of growth responses upon the encounter of obstacles, bends, or constrictions (Agudelo *et al*., 2012). The dual-flow RootChip was the first device for primary roots of Arabidopsis seedlings that demonstrated the possibility to use micropillars as structural guides for root growth direction in a bi-laminar flow (Stanley *et al*., 2018) (Fig. 3A). Another device adapted for taproots, a microfluidic environment for root colonization studies, displayed not only the use of pillars as a simulation of a soil matrix but also the possibility for sub-compartmentalization along the microfluidic device (Aufrecht *et al*., 2018). Similarly, the introduction of pillars was seen in the Ecosystem Fabrication (EcoFab) concepts, which originally introduced sand or small-sized soil particles (Gao *et al*., 2018; Jabusch *et al*., 2021) (Fig. 3B). The employment of granular media algorithms trained on native sandy soils was then utilized first in the field of soil microecology to design soil matrix-like masks for poly(dimethylsiloxane) (PDMS) devices, that were initially used to track biofilm development influenced by pore hydrodynamics (Aufrecht *et al*., 2019). Algorithm-generated soil-like matrices could thus show the role of rheotaxis, current-oriented motility to the present fluid flow, which determined the initial adherence of bacterial cells by influencing their translocation. In a rather reductionist approach, different types of geometric structures present in soil were integrated on-chip to track hyphal growth, particle transportation, and foraging behaviour of fungi, bacteria, and protists (Mafla-Endara *et al*., 2021) (Fig. 3C). The transfer of algorithm-based designs of soil-mimicking PDMS matrices was lastly seen with the development of rhizosphere-on-a-chip, which utilized a similar approach to that of Aufrecht *et al*. (2018) for a liquid-filled PDMS matrix for the growth of *Brachypodium distachyon* (Aufrecht *et al*., 2022) (Fig. 3E). In the rhizosphere-on-a-chip, Aufrecht *et al.* could display that a granular environment leads to the constitution of specific chemical niches in surrounding substrates, in this case through the enrichment of amino acids by exudation. Asymmetric concentrations of carbon compounds could further be driving factors of the spatial distribution of interacting bacteria and chemical profiles of the surrounding soil. The rhizosphere-on-a-chip device therefore highlights the potential of soil-mimicking devices to disentangle the interaction between heterogeneous granular matrices and the root’s activity, while retaining a controlled experimental platform and visual access.

Mechanical stimuli are not the only physical factors shaping the interaction in the rhizosphere. Similarly, soil displays dynamic temperature gradients, with the upper soil layers following variations in solar radiation and deeper layers exhibiting a more constant temperature (Singh and Sharma, 2017). Temperature gradients have already been achieved in microfluidic devices adapted for chemical research utilizing asymmetric hot and cold fluid streams (Mao *et al*., 2002). To study the cold-mediated inhibition of Arabidopsis seed germination, a PDMS-based phenotyping system was developed by pairing an inclined PDMS block with a thermo-electric cooling plate, generating a temperature gradient in the growth channels engraved into the PDMS block (Wang *et al*., 2017).

The representation of soil structures on chips and in microdevices has advanced not only through the development of transparent soil but also through the algorithm-assisted design of PDMS masks to reflect granulated patterns of soil. In this way, spatially heterogeneous environments can be created while preserving microscopic access.

### Chemical heterogeneity

The chemical heterogeneity of soil originates from the mineral and organic composition of the soil matrix, as well as from the influence of the organisms present and the plant root itself, releasing bioactive compounds and organic matter. The chemical profile of the surrounding soil matrix includes compounds acting as nutrients, stress elicitors, and signal molecules including phytohormones and volatiles modulating cellular functions. These conditions display spatiotemporal variation and challenge the root system with locally asymmetric conditions, which raises the question of how local stimuli and temporal dynamics affect the overall acclimation of root systems.

The development of the RootChip, a fully integrated system with on-chip cultivation of multiple seedlings and micromechanical valves for perfusion control, was aimed at the temporal dynamics of growth conditions. Rapid exchange of the root microenvironment was achieved by applying pulsed treatments to *A. thaliana* roots in a linear channel with a root-circumferential laminar flow (Grossmann *et al*., 2011). This system was used for live-cell imaging under pulsed treatment with glucose and galactose, for which transport kinetics could be determined using genetically encoded biosensors. Pulsed treatments with short delay times are a particular strength of microfluidic perfusion systems due to the small dead volumes. The rapid switching of solutions was critical in a biosensor-based quantitative assessment of intracellular Zn^2+^ concentration in Arabidopsis vacuoles (Lanquar *et al*., 2014), the characterization of the biosensors ABACUS for abscisic acid (ABA) (Jones *et al*., 2014), and CerTN-L15 for calcium *in planta* (Denninger *et al*., 2014), or the discovery of rapid, non-genomic growth inhibition upon auxin application (Fendrych *et al*., 2018). The simplified version RootChip-8S, replacing the on-chip valve system with external flow control (Denninger *et al*., 2019; Guichard *et al*., 2020), has contributed to a wider dissemination of the technique and enabled, among others, the testing of computational models of mechanisms underlying gibberellin gradients in growing roots (Rizza *et al*., 2021). Recently, pulsed perfusion with concentrated osmolytes enabled the quantitative description of cell wall elasticity in the root elongation zone and helped discover a cytokinin-dependent mechanism that modulates cell wall mechanics and thus limits cell expansion and promotes cell differentiation (Liu *et al*., 2022).

Besides temporal control over the chemical conditions, spatial heterogeneity or even localized treatments are an important, yet more challenging aspect of approaching soil-emulating growth conditions. Laminar flow, the predominant flow regime in microfluidic environments, can help to create gradients or asymmetric conditions with sharp boundaries (Stanley *et al*., 2018; Convery and Gadegaard, 2019) (Fig. 3A).

Therefore, the first functionality integrated into a root-adapted microfluidic device was the localized treatment of roots. The device design by Meier and colleagues used three parallel laminar streams to allow a local application of a liquid treatment to an *A. thaliana* root segment of 10–800 µm (Meier *et al*., 2010). A central liquid stream carrying an auxin derivative was focused perpendicular to the root by two lateral streams that carried an auxin transport inhibitor, triggering localized emergence of root hairs at the treated site. While truly pioneering plant microfluidics, the downsides of this early device included the potentially invasive manual mounting of a single root within the treatment chamber and the limited range within which the focused stream could target the root.

The dual-flow RootChip aimed at spatial asymmetry by generating a bi-laminar flow laterally of the centrally confined root via two separate inlets (Stanley *et al*., 2018). This setup enabled the study of asymmetric acclimation of root hair growth to locally different concentrations of phosphate. The experiments demonstrated that root hairs are able to rapidly respond to local nutrient conditions with a non-genomic, cell-autonomous regulation of growth rate and duration.

Previously mentioned approaches focused on the taproot system of *A. thaliana*, but proved incompatible with the fibrous root systems of monocots. An approach to adapt such microdevices to monocots can be seen in the petaloid chamber root growth chip for the cultivation of the fibrous root system of *Oryza sativa*, with five separate chambers connected to a central column holding the seedling (Chai *et al*., 2019). Each chamber could be separately filled with either a solid or liquid medium, allowing the generation of independent conditions for each root. This setup was utilized to assess the root system architecture if challenged with differing degrees of water availability by polyethylene glycol (PEG) 6000 treatment, in which the roots primarily elongated in chambers of higher water availability.

The RootTrapr, designed based on the Ecosystem Fabrication setup (Gao *et al*., 2018), could assess the influence of the defence-stimulating elicitor chitosan on *Cannabis sativa* and allowed the display of an ABA-related reduction in lateral root formation, as well as a multi-omics analysis of the plant’s response (Suwanchaikasem *et al*., 2022).

Microfluidic chips have not only enabled the assessment of the effects of chemical stimulants and asymmetric chemical conditions but can also be utilized to sample and assess chemical changes originating from the plant’s activity. In general, microfluidic platforms can be used as an alternative cultivation platform for the *ex situ* analysis of metabolites and exudates. For example, the EcoFab was used to grow *B. distachyon* in synthetic media and sterile soil extract, during which root phenotypes were captured throughout the growth period. This enabled a metabolic analysis of phosphate content and exudate profile (Sasse *et al*., 2019), revealing distinct metabolic responses to the complex soil extract and increased root hair length. *In situ* analysis on-chip has also been achieved: a microfluidic cultivation platform for *O. sativa* was fitted with an integrated nutrient sensor, which allowed the non-invasive tracking of nitrate and phosphate uptake from the medium for up to 15 d (Jiang *et al*., 2017). Further, a microfluidic imaging chamber confining a mature root of *O. sativa* based on the readout of a redox-sensitive dye allowed the display of a day and night cycle-dependent production of reactive oxygen species (ROS) (Dai *et al*., 2022) (Fig. 3D). Lastly, these diurnal patterns of ROS production were linked to an interplay in the rhizosphere between bacterial respiration and oxygen release by the root system.

While these systems were confined to specific nutrients and chemical compounds, a further advancement was the direct coupling of LC/MS to microfluidic habitats for root growth. This was achieved by integrating nanoporous polyester track-etched (PETE) membranes at specific positions into a microfluidic growth system for a wheat seedling’s root, which allowed metabolic exchange with an integrated microfluidic sampling system (Patabadige *et al*., 2019). The setup enabled dynamic tracking of root exudation at two spatially separated positions, and the detection of saccharides and amino acids characteristic of root exudates. This approach was further advanced by coupling a soil-like PDMS matrix on-chip for growing *B. distachyon* and a PETE membrane to liquid micro-junction surface sampling probe mass spectrometry (LMJ-SSP-MS), which allowed the capture of the spatial distribution of amino acid exudation at a high resolution (Cahill *et al*., 2020; Aufrecht *et al*., 2022) (Fig. 3E). LMJ-SSP-MS itself is a microfluidic technique, that was designed to allow chemical sampling on a running microfluidic biology-on-a-chip device; in this way a liquid junction generated between the sampling device and the porous membrane is utilized to extract nanolitre to microlitre volumes out of the biology-on-a chip device (Cahill *et al*., 2020). As such, microfluidic technology cannot only aid the simulation of soil-like conditions but also expand the available sampling and analytical toolset.

### Biotic heterogeneity

Soil is a biodiverse and densely populated ecosystem, enabled by the variety of available chemical substrates and structures. A single gram of soil contains on average 10^10^ bacterial cells, 10^2^–10^4^ cells of eukaryotic protists, 10–20 m of fungal hyphae, and ~10 nematodes (Roesch *et al*., 2007; Geisen *et al*., 2018; Das and Dash, 2019; Lehmann *et al*., 2020; van den Hoogen *et al*., 2020). The various species can act as pathogens, parasites, commensals, and mutualists, while also providing essential ecosystem services by driving nutrient cycling and mineralization, and influencing soil structure (Forster, 1990; Adl and Gupta, 2006; Figueiredo *et al*., 2016; Seppey *et al*., 2017). The plant’s innate immune system, cell wall composition, and deposition of organic matter further filter the soil biota which are present into a specific rhizosphere community, that characterizes the root-influenced soil (Durán *et al*., 2018; Canarini *et al*., 2019; Knights *et al*., 2021).

On the smallest scale, and most abundant, are bacterial species (Osiro *et al*., 2012). Bacterial association in the rhizosphere can range from structured communities filtered into the rhizosphere to biofilm coverage of the root’s surface, and endophytes invading root tissues (Bai *et al*., 2015; Deng *et al*., 2019; Knights *et al*., 2021) and also being widespread causes of plant disease (Huang *et al*., 2020). Microfluidic bioreactor designs have been deployed in bacterial research since the early 2000s, independently of plant systems (Heo *et al*., 2003; Balagaddé *et al*., 2005; Grünberger *et al*., 2012; Burmeister *et al*., 2019). Using the above-described dual-flow-RootChip, the micropillars guiding the root proved suitable for trapping green fluorescent protein (GFP)-expressing *Pseudomonas fluorescens* along the Arabidopsis root (Stanley *et al*., 2018). A device specifically targeted at root–microbe interaction is the Tracking Root Interaction System (TRIS), which was used to perform live-cell imaging of the first 6 h of colonization by *Bacillus subtilis* NCIB 3610 (Massalha *et al*., 2017). This setup enabled access to spatio-temporal patterns of colonization and could identify the elongation zone as an area of initial association with the root. In addition, a basic consortium of *E. coli* and *B. subtilis* was tested and could display displacement of *E. coli* by *B. subtilis* from the root’s surface. Similarly, a microfluidic device was employed to track colonization by *P. fluorescencs* SWB25 on *Populus tremoloides* roots and *B. subtilis* NCIB 3610 on *O. sativa* roots in a setup termed an RMI (root–microbe interactions) chip (Noirot-Gros *et al*., 2020) (Fig. 3F). While species of the *B. subtilis* species complex and pseudomonads are regularly isolated from the rhizosphere and include agronomically relevant species (Fan *et al*., 2017), these are only one of many taxa represented in the rhizosphere. The next step in reflecting structured communities of the rhizosphere is the transfer of native rhizosphere strains. As such, endophytic and rhizosphere-associated strains isolated from the *P. tremoloides* microbiome were assessed for their spatiotemporal dynamics of root colonization on *A. thaliana* in a microfluidic habitat, which over the course of a 4 d co-cultivation period allowed the correlation of inoculum density with the formation and size of endophytic microcolonies (Aufrecht *et al*., 2018). With comprehensive collections of rhizosphere microbiota strains of *A. thaliana* and further species such as *Lotus japonicus*, the next frontier is the integration of simplified but taxonomically representative synthetic communities into root-adapted microfluidics (Bai *et al*., 2015; Wippel *et al*., 2021); this is currently preceded by multi-species experiments performed in microfluidic setups outside of plant research (Alnahhas *et al*., 2019).

Eukaryotic species including oomycetes, fungi, nematodes, and various taxa of protists are another major constituent of the soil biota and source of plant-protective and pathogenic species (Richards *et al*., 2006; Giraldo and Valent, 2013). Zoospores of the oomycete *Phytophthora sojae* and sugar beet nematode were the first pathogenic species combined with *A. thaliana* in an early microfluidic setup, the plant-in-chip device (Parashar and Pandey, 2011). The plant-in-chip targeted the establishment of pathogen–root interaction and could thus display the 1–2 h period in which nematodes were attracted to the root and then enabled the invasion of root tissue 2–3 d post-inoculation to be followed. Zoospores were found to initially accumulate close to the root tip around 2 hours post-inoculation (hpi), forming a cluster, from which hyphae grew out and penetrated the plant’s tissue from 6 hpi onward (Parashar and Pandey, 2011). These experiments displayed the potential of microfluidic habitats to target, in particular, early stages of association with eukaryotic plant pathogens. For fungal species, a variety of microfluidic devices have been published (Richter *et al*., 2022), while for protozoan species the field of microfluidic soil-like approaches is just emerging (Gaines *et al*., 2019). A multi-species integration including protists, fungi, and nematodes in a soil-like structured environment assessed the dynamics of soil–structure–organism interaction (Mafla-Endara *et al*., 2021) (Fig. 3F). The establishment of such multi-species or multi-kingdom setups in soil-like microdevices with plant roots has yet to be shown.

## Conclusion and outlook

Root–soil interactions result from multi-scale processes, with length scales ranging from micrometres to centimetres, and time scales ranging from milliseconds to days. Processes on larger length and time scales are experimentally accessible with *in situ* phenotyping setups such as rhizotrons, the GloRoot platform, or µ-CT (Rellán-Álvarez *et al*., 2015; Atkinson *et al*., 2019). However, these systems are less suitable to address the micrometre-scaled, rapid dynamics that are a major constituting factor of root–soil interactions. Due to their versatility, microfluidic devices facilitate the simulation of specific environmental conditions, provide access to rapid micrometre-scaled processes, and allow, dependent on the device and species, high-resolution imaging from days to weeks (Stanley *et al*., 2018; Sasse *et al*., 2019). Consequently, microfluidics can contribute to a broad range of research areas from prokaryotic interactors to root system architecture (Table 1).

Root microdevices have greatly advanced since the beginning of the 2010s in reconstituting soil-like conditions. Structural scaffolds have moved from linear unstructured chambers and channels to PDMS matrices based on the particulate structure of native soils (Grossmann *et al*., 2011; Gao *et al*., 2018; Stanley *et al*., 2018; Jabusch *et al*., 2021; Mafla-Endara *et al*., 2021; Aufrecht *et al*., 2022). Furthermore, the range of species has greatly expanded from the dicot *A. thaliana* to a variety of monocots including *O. sativa*, *B. distachyon*, wheat, and non-model species such as *C. sativa* (Chai *et al*., 2019; Sasse *et al*., 2019; Aufrecht *et al*., 2022; Suwanchaikasem *et al*., 2022).

Progress in accessing chemical interactions with the rhizosphere has primarily been made by including advanced microfluidic chemical analysis tools and sensors into microfluidic cultivation chambers, enabling *in situ* analysis at high spatio-temporal resolution (Patabadige *et al*., 2019; Cahill *et al*., 2020; Aufrecht *et al*., 2022), thus offering novel possibilities to the study of exudation and signalling. As such, root-adapted microdevices paired with advances in visualization of morphological changes of tissues, namely morphodynamics, could further underline the integration of environmental conditions into the reshaping of root architecture and the transition between local and systemic responses based on tracking of signalling dynamics. The capabilities of *in situ* analysis of the plant’s status and also environmental properties could be further supported by the integration of bioelectronics into PDMS-based microdevices including nutrient sensors, spectrometric readouts, and measurements of electric field changes, but also the capabilities of localized stimulation (Dufil *et al*., 2022). The coupling of bioelectronics and microfluidics in fully integrated, miniaturized arrays of lab-on-a-chip technology will probably reduce the need for external analysis hardware.

Microbial species define the rhizosphere and contribute to plant performance and resilience. Microbial interactors, mostly bacterial species, have therefore also been included in root-adapted microfluidics (Massalha *et al*., 2019; Noirot-Gros *et al*., 2020; Jabusch *et al*., 2021), and have also expanded from model species such as *E. coli* and *B. subtilis* to native rhizosphere strains (Aufrecht *et al*., 2018). Whether a full reflection of taxonomically diverse rhizosphere communities, as seen in the usage of synthetic communities in the study of rhizosphere microbiology (Bai *et al*., 2015; Thiergart *et al*., 2020; Wippel *et al*., 2021), can be established in microdevices, is yet to be shown. Despite the representation of bacterial species, filamentous eukaryotes containing major plant pathogens and symbionts have not seen a similar representation in microdevices, even in early tests with oomycetes (Parashar and Pandey, 2011). Fungal pathogens and symbionts in particular would be prime targets for the future direction of soil-like microdevices similar to the root–bacteria interaction assessing temporal dynamics, potential influences of environmental stress, and utilizing advances in highly resolved chemical analysis to track nutrient trading during symbiosis.

Despite a wide range of potential applications, the utilization of microfluidics in plant research is still limited as only a few designs are used repeatedly, notably the EcoFab and RootChip devices (Denninger *et al*., 2014, 2019; Jones *et al*., 2014; Lanquar *et al*., 2014; Fendrych *et al*., 2018; Stanley *et al*., 2018; Rizza *et al*., 2019; Sasse *et al*., 2019; Hani *et al*., 2021; Kuang *et al*., 2022; Liu *et al*., 2022; Suwanchaikasem *et al*., 2022). Conventional methods of producing microfluidics, namely soft lithography, are a limiting factor by being costly and slow, and therefore not allowing rapid prototyping and in-house manufacturing. The integration of 3D printing into microfluidic manufacturing since the 2010s is moving this barrier (Ho *et al*., 2015; Waheed *et al*., 2016). The EcoFab concept builds on 3D-printed casting moulds for PDMS, as also the multi-petaloid chamber, and even fully 3D-printed microdevices have been tested in association with plants (Gao *et al*., 2018; Chai *et al*., 2019; Guichard *et al*., 2021, Preprint; Moussus and Meier, 2021). While, 3D-printing techniques provide access to rapid prototyping and enable full in-house production capabilities for many research labs, resolution is still a restriction for a full replacement of soft lithography. However, through the use of high-resolution production techniques such as two-photon or triplet–triplet annihilation polymerization, submicrometre resolutions could become achievable and affordable in the near future (Limberg *et al*., 2022).

In addition to the complexity of production, the usability of microdevices can also depend on active liquid handling capabilities. Consequently, the complexity of devices has reduced through their design cycle. For example, the RootChip device transitioned from an on-chip liquid handling to a linear channel design with external liquid handling (Grossmann *et al*., 2011; Stanley *et al*., 2018; Guichard *et al*., 2020). Similarly, the EcoFab device and its associated community have focused on a modular design and components easily adapted to different experimental questions (Gao *et al*., 2018). The complexity of the readouts leads to a further drawback, by restricting the throughput of microfluidic devices. Most devices display sample numbers in the range of 5–20, with only few exceptions exceeding that (RootArray, PlantArrayChip) (Table 1). Studies on root–environment interactions using microfluidics face the challenge of achieving a modular reconstruction of soil complexity towards an idealistic soil-on-a-chip device while ensuring sufficient throughput, accessibility, and ease of use.

Although microfluidic devices have helped improve controllability compared with traditional environments such as agar plates or hydroponics, they still suffer from common issues. One example is the illumination of the root system during growth, for which a range of side effects have been reported including changes in nutrient uptake, biotic interactions, root exudation, and root development (van Gelderen *et al*., 2018; Cabrera *et al*., 2022). To avoid inherent biases for the study of roots under light exposure, several studies have included light shielding of agar plates (Xu *et al*., 2013; Silva-Navas *et al*., 2015). Such concepts have also been developed for microfluidic devices, such as a shielding insert for the RootTrapr device (Suwanchaikasem *et al*., 2022). Similarly, for certain stresses such as drought, only proxies can be utilized in a liquid-based system, for example the application of osmolytes such as PEG, mannitol, sorbitol, or salt. Such proxies lower the water activity but also differ in their specific effect and do not constitute the full complexity of drought stress under field conditions (Dubois and Inzé, 2020). Consequently, standardizing best practices between traditional cultivation systems and microfluidic devices will be a necessary step for the field of root–environment studies.

Finally, beyond their application for root–environment studies, the versatility of structured microdevices has the potential to help uncover mechanisms underlying the integration of sensory information over space and time in root growth under non-uniform conditions (Meroz, 2021).
